# Conservation, Compensation, and Evolution of N-Linked Glycans in the HIV-1 Group M Subtypes and Circulating Recombinant Forms

**DOI:** 10.5402/2012/823605

**Published:** 2012-12-19

**Authors:** Simon A. Travers

**Affiliations:** Medical Research Council Unit for Bioinformatics Capacity Development, South African National Bioinformatics Institute, University of the Western Cape, Private Bag X17, Belville 7535, South Africa

## Abstract

The “glycan shield” exposed on the surface of the HIV-1 gp120 *env* glycoprotein has been previously proposed as a novel target for anti-HIV treatments. While such targeting of these glycans provides an exciting prospect for HIV treatment, little is known about the conservation and variability of glycosylation patterns within and between the various HIV-1 group M subtypes and circulating recombinant forms. Here, we present evidence of strong strain-specific glycosylation patterns and show that the epitope for the 2G12 neutralising antibody is poorly conserved across HIV-1 group M. The unique glycosylation patterns within the HIV-1 group M subtypes and CRFs appear to explain their varying susceptibility to neutralisation by broadly cross-neutralising (BCN) antibodies. Compensatory glycosylation at linearly distant yet three-dimensionally proximal amino acid positions appears to maintain the integrity of the glycan shield while conveying resistance to neutralisation by BCN antibodies. We find that highly conserved clusters of glycosylated residues do exist on the gp120 trimer surface and suggest that these positions may provide an exciting target for the development of BCN anticarbohydrate therapies.

## 1. Introduction

The envelope gene of human immunodeficiency virus type 1 (HIV-1) encodes a gp160 precursor that is cleaved to form gp120 and gp41 that exists as a trimer on the surface of a HIV virion and is responsible for host cell recognition and binding. As the envelope protein moves through the endoplasmic reticulum, N-linked glycans are added to aid correct folding and processing of the protein [1–3]. The gp120 protein is one of the most heavily known glycosylated proteins [3–5]. The carbohydrates present on gp120 are created by the host cell and, as such, are recognised as immunologically “self” by the host immune system. Studies have shown that the “glycan shield” bound to gp120 can prevent neutralisation of the virus by antibodies [6–13]. It has been suggested that lowly glycosylated viruses may be replicatively fitter and are thus selected early on in infection with glycosylated viruses only being selected for following the activation of the host humoral immune response [14–18]. This trend does not occur in all cases; however, it has been suggested that it occurs more frequently in particular viral subtypes [17, 19]. 

Domains on gp120 responsible for receptor binding and trimer interactions tend to exhibit low levels of glycosylation resulting in the designation of three domains within gp120: the neutralizing face, the nonneutralizing face, and the silent face [20–22]. The neutralizing face comprises the receptor-binding sites while the non-neutralizing face contains epitopes that are accessible to neutralizing antibodies in monomeric gp120 but which are hidden in the gp120-gp41 trimer. The highly glycosylated domain has been termed the silent face given that is immunologically “self” to the host immune system. It has been suggested, however, that the highly conserved glycans on the gp120 surface may, themselves, provide an ideal target for neutralizing antibodies [18, 23]. In fact, the neutralizing antibody 2G12 binds to a well-defined epitope comprising solely of N-linked glycans bound to the gp120 surface [24–26]; however 2G12 has been shown to have varying efficacy for different subtypes and is particularly ineffective against subtype C and CRF01_AE [27–29]. More recently, a number of studies have isolated BCN antibodies whose activity appears to be highly dependent on the presence of glycosylation at a number of positions on the gp120 trimer, particularly position 332 [30–33]. Work has also shown that lectins isolated from various sources exhibit antiviral activity by interacting with the carbohydrates bound to gp120 and, thus, block cell-to-cell contact between gp120 and the host cell thereby inhibiting cell binding and fusion [34–39]. Similarly, Balzarini and colleagues have shown that Pradimicin A, an antifungal antibiotic, displays properties that inhibit virus entry into host cells [40]. Due to the high degree of glycosylation of gp120, targeting these carbohydrates means that there are multiple targets available, and the emergence of resistance to such anticarbohydrate therapies will most likely involve the removal of multiple glycosylated sites thereby exposing the surface of the virus to neutralizing antibodies [40–42].

HIV-1 group M, which is responsible for the vast majority of HIV infections worldwide, exhibits incredibly high levels of genetic diversity, and phylogenetic analysis has determined a number of major clades/subtypes within the group M phylogeny (designated A-D, F-K) [43]. While subtype B predominates in North America and Europe [44], subtype C accounts for greater than 50% of worldwide infections [45]. Recombination occurs frequently between HIV-1 group M subtypes with established recombinants described as circulating recombinant forms (CRFs). These CRFs now account for as much as 18% of the worldwide infections [46, 47] with CRF01_AE and CRF02_AG representing the most prevalent forms in Southeast Asia and West/West Central Africa, respectively [48–52]. In light of numerous studies highlighting the vast potential of anti-carbohydrate therapies in HIV treatment, it is important that we fully understand the complexities and dynamics of N-linked glycosylation in the most predominant strains of HIV currently circulating within the worldwide population. 

Previous work has examined glycosylation patterns within a selection of sequences representing the HIV-1 group M subtypes and CRFs and concluded that despite the extreme genetic variability between the HIV-1 group M subtypes and CRFs, the patterns of glycosylation are essentially conserved between them [53]. Poon and colleagues studied the evolutionary interactions between N-linked glycosylation proposing that mutual exclusion of glycosylation occurs between colocalized glycans while mutual dependency occurs between structurally distant glycans [54]. Here, we have expanded upon these previous studies by looking at the conservation and strain-specific patterns of N-linked glycosylation across all available sequences from full-length genomes representing the HIV-1 group M subtypes and CRFs. We examine the prevalence of the 2G12 epitope and identify mutations that cause loss of sensitivity to 2G12 neutralisation yet still maintain the protective glycan shield on the gp120 trimer. Further, we identify clusters of glycosylated positions that are highly conserved across the entire spectrum of the HIV-1 group M subtypes and CRFs that show potential as targets for BCN carbohydrate-binding therapies.

## 2. Materials and Methods

### 2.1. Data Selection

All available full-length genomes representing the HIV-1 group M subtypes and circulating recombinant forms (CRFs) were retrieved from the Los Alamos National Laboratory (LANL) HIV Sequence Database (http://www.hiv.lanl.gov/). Full genomes were used in order to avoid the possibility of including intersubtype recombinants in the analysis. Using the patient and isolate annotation, where available, multiple sequences representing single individuals were removed to ensure that the dataset representing each subtype/CRF was nonredundant. Subtypes and CRFs with significant numbers of representative sequences were selected for subsequent analysis (subtypes A (*n* = 73), B (*n* = 161), C (*n* = 385), D (*n* = 48), CRF01_AE (*n* = 90), and CRF02_AG (*n* = 38), see Supplementary Table 1 Supplementary Material available online at doi: 10.5402/2012/823605). For all selected subtypes/CRFs, the fragment of the *env* gene encoding gp120 was excised from the full genome, and all sequences were manually aligned using MacClade [55]. Regions of the variable loops that proved difficult to align were excluded in order to avoid false positives due to alignment ambiguities. The final dataset comprised 820 taxa and was 2394 nucleotides (798 amino acids) in length.

### 2.2. Glycosylation Prediction

Potential glycosylated positions (sequins) were predicted by scanning the amino acid sequences to identify the presences of NX[ST] motifs where X is any amino acid. Studies have shown that NXT motifs where X is P and NXS motifs, where X is P, W, D, E, or L are poor oligosaccharide acceptors and are less likely to be glycosylated [56], and, thus, we discounted these motifs as potential glycosylated positions. Any sequon utilizing the NNTT motif or NNSS motif was categorised as a single glycosylation event as steric occlusion means that glycosylation at both adjacent positions is highly unlikely. To enable direct comparisons between subtypes within this study and between other studies all results presented here correspond to HXB2 gp120 amino acid numbering. Positions predicted as glycosylated in more than 75% of sequences from at least one of the subtypes/CRFs were identified and, as such, represent highly conserved glycosylated positions.

### 2.3. Glycosylation within Variable Loops

Due to their high sequence diversity, the variable loops could not be aligned directly to each other. For each gp120 sequence, each of the variable loops was extracted and analysed independently of all other sequences without prior alignment. Therefore, while direct comparison between specific glycosylated positions was not possible, identification of the number of predicted glycosylated positions in each sequence as well as correlation of these numbers with variable loop length was performed. The variable loops were defined according to the information contained on the LANL HIV Sequence Database with V1/V2 ranging from 131C to 195S, V3 from 296C to 330H, V4 from 385C to 418P and V5 from 461S to 470P (HXB2 numbering).

### 2.4. Gp120 Structure Analysis

3D structural analysis was performed using the gp120 V3 loop-containing structure covering residues 84V-492E [57]. An *in silico* approach was used to add sugars to the gp120 structure using GlyProt [58] with the type of carbohydrate added, where available, having been previously determined [59]. It must be noted, however, that these carbohydrate types are only used for representation purposes and there is a strong likelihood that carbohydrate type may differ between subtypes/CRFs. The gp120 structure corresponds to a subtype B virus [57] and, where necessary, the amino acid sequence of the structure was mutated using MacPyMol, the Macintosh version of PyMol (http://pymol.sourceforge.net/). 

## 3. Results

**Figure 1:**
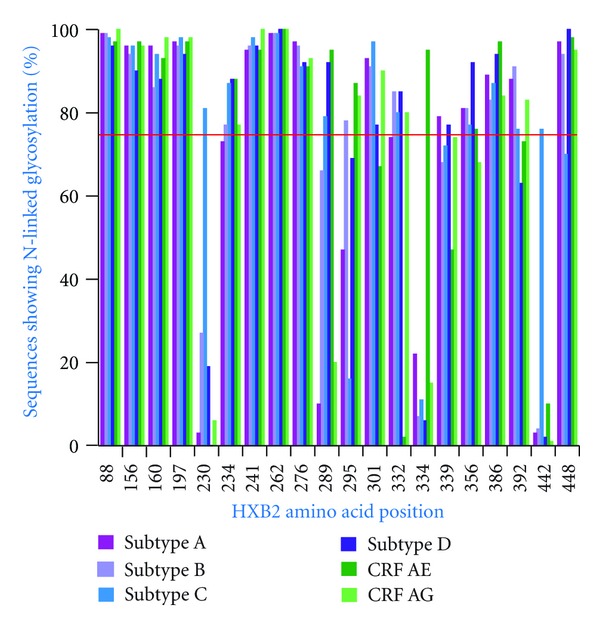
Prevalence of glycosylation at selected positions. The relative frequency of glycosylation between subtypes/CRFs at positions where at least one subtype/CRFs shows high levels of glycosylation. Positions where greater than 75% of sequences were predicted as glycosylated for at least one subtype/CRF are shown.

In general, the average numbers of predicted glycosylated positions are relatively conserved across the HIV-1 group M subtypes/CRFs showing an average of 15-17 glycosylated positions in gp120. In order to identify sequons that are highly conserved within and/or between subtypes, all positions where sequons were identified in greater than 75% of sequences for at least one subtype/CRF were plotted (Figure 1). Positions 88N, 156N, 160N, 197N, 234N, 241N, 262N, 276N, 301N, 356N, 386N, 392N, and 448N all showed extremely high levels of conservation of glycosylation across all subtypes/CRFs studied. A number of subtype-specific patterns were identified, however, with 230N and 442Q showing high levels of predicted glycosylation within subtype C (81% and 76% of subtype C sequences, resp.) when compared to all other subtypes/CRFs (Figure 1). Also, position 295N is predicted as being glycosylated in a smaller number of subtype A and C sequences (47% and 16% resp.), when compared with the other subtypes/CRFs (Figure 1). Position 334S is predicted as glycosylated in 95% of CRF01_AE sequences with it only being observed as glycosylated in between 6% and 22% of sequences representing the other subtypes/CRFs (Figure 1). CRF01_AE also shows very low predicted levels of glycosylation at position 332N while between 74% and 85% of sequences representing the other subtypes/CRFs are predicted as glycosylated at 332N.

### 3.1. Variable Loop Glycosylation

As a number of domains within the variable loops were impossible to align with confidence, these had been excluded from the analysis of glycosylation in gp120 in all subtypes and CRFs. For each sequence representing the subtypes/CRFs, glycosylation within the variable loops was studied without prior alignment. While glycosylation at specific positions cannot be directly compared within or between subtypes/CRFs, these results do give us an overview to the levels of glycosylation within the variable loops. The average length of the V1/V2 loop for each of the subtypes/CRFs ranged between 65 and 69 (Table 1) amino acids suggesting that V1/V2 length is relatively conserved between the majority of virus strains in all subtypes/CRFs. Within the V1/V2 domain there was a large spread of predicted glycosylated positions ranging from one to eleven positions with a mean of six glycosylated positions for all of the subtypes/CRFs (Table 1). Interestingly, regression analysis suggests that as the length of the V1/V2 variable loop increases so does the number of glycosylated positions suggesting that the two are dependent (data not shown). For all subtypes/CRFs, the V3 loop averaged 34 amino acids in length and had a highly conserved glycosylation pattern with the vast majority only predicted as glycosylated at one position (301N) and a small number of sequences from B (2%), C (2%), AE (1%), and AG (2%) being predicted as glycosylated at two positions within V3 (Table 1). Both V4 and V5 show conserved patterns of glycosylation between the subtypes/CRFs with an average of 4-5 glycosylated positions for V4 and 1-2 for V5 (Table 1). 

### 3.2.  2G12 Conservation

The 2G12 neutralizing antibody targets carbohydrates bound to the gp120 surface [24], yet it has been shown to have low efficacy against subtype C and CRF01_AE [27, 28]. It has been identified that the 2G12 epitope is centred around positions 295N, 332N, and 392N while peripheral glycosylated positions 339N, 386N, and 448N are not critical for (but play a role in) 2G12 binding [25, 26]. Previous work has suggested that glycosylation at the core sites of the 2G12 epitope is well conserved, with the exception of 295N in subtype C [53]. In this study, however, we find that glycosylation of all of the core sites of the 2G12 epitope (295N, 332N, and 392N) is not particularly well conserved ranging from 1% of CRF01_AE sequences to 61% of subtype B sequences (Table 2). Subtype C and CRF01_AE, in particular, show very low conservation of glycosylation at the 2G12 epitope core, 11% and 1%, respectively (Table 2). In subtype C the reason glycosylation at the core positions of the 2G12 epitope is so poorly conserved is because position 295N is only predicted as glycosylated in 16% of subtype C sequences, while for CRF01_AE position, 332N is only glycosylated in 2% of sequences (Figure 1). When the proposed peripheral positions (339N, 386N, and 448N) are included, the conservation of glycosylation of the extended 2G12 epitope drops to 1% for CRF01_AE up to 36% for subtype B (Table 2).

### 3.3. Conservation of Glycosylation at Multiple Positions

**Figure 2:**
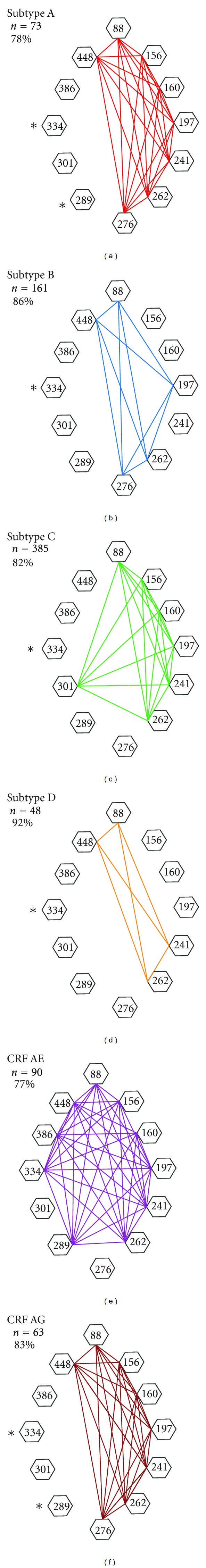
Networks of positions with highly conserved glycosylation. Networks of highly conserved glycosylated pairs identified in each HIV-1 group M subtype/CRF. Pairs where greater than 90% of sequences exhibit glycosylation at both positions are joined by a line. In all cases, the networks are mutually dependent such that each position in the network is seen as highly dependent with every other position in the network. The number of sequence in each dataset is represented as *n* while the percentage of sequences glycosylated at all of the positions in the network for the respective subtype/CRF is also shown. Positions that are predicted as N-linked glycosylated in less than 25% of sequences for a particular subtype/CRF are marked with a ∗.

In order to examine the levels of conservation of glycosylation within the subtypes/CRFs studied, we identified any glycosylated pairs where both positions were predicted to be glycosylated together in greater than 90% of sequences representing a particular subtype/CRF. The number of highly conserved glycosylated pairs ranged from 16 (subtype D) to 44 pairs (CRF01_AE; Supplementary Table 2) with only five pairs (88 and 241, 88 and 262, 197 and 262, 241 and 262, and 262 and 276) observed as conserved over all the subtypes/CRFs. Interestingly, all subtypes/CRFs showed networks where glycosylation of all residues in the network is highly conserved with all other members in the network (Figure 2). CRF01_AE shows the strongest network of dependency with 11 positions all showing high conservation of glycosylation among them while subtype D only shows a conserved network containing four residues (Figure 2). Similarly, each subtype/CRF shows a number of highly conserved pairs which do not comprise the core conserved network (Supplementary Table 2).

## 4. Discussion

It has been previously suggested that the glycan shield covering the HIV gp120 trimer exposed on the surface of a virion may provide a novel target for anti-HIV treatments [18, 23, 40]. While the targeting of these glycans provides an exciting prospect in HIV treatments, very little is known about the conservation and dependencies of glycosylation within and between the various HIV-1 group M subtypes and circulating recombinant forms. Here, we have performed a comprehensive study examining the glycosylation patterns present in all major circulating forms of HIV-1. 

Previous work examining glycosylation patterns in a subset of HIV-1 group M sequences suggested that despite the extreme genetic variability between the HIV-1 group M subtypes and CRFs, the patterns of glycosylation are essentially conserved between them [53]. We have observed such a pattern at some position, with 13 amino acid positions in particular exhibiting high levels of predicted glycosylation across all subtypes/CRFs studied (Figure 1). However, we do find that a number of amino acid positions show strong subtype/CRF-specific glycosylation patterns (Figure 1) and that these positions form an essential part of the epitopes for many BCN antibodies [25, 26, 30, 31, 33].

### 4.1. Conservation of the Epitopes of BCN Antibodies

Of all of the neutralising antibodies that target the glycan shield, the epitope for 2G12 is the most well defined and is centred around glycosylation of positions 295N, 332N, and 392N in gp120 while peripheral glycosylated positions 339N, 386N, and 448N are not critical for (but play a role in) 2G12 binding [25, 26]. Previous work has suggested that glycosylation at the core sites of the 2G12 epitope is well conserved, with the exception of 295N in subtype C [53]. By studying all available *env* sequences, we find, however, that glycosylation of the core 2G12 epitope (295N, 332N, and 392N) is, in fact, poorly conserved across all HIV-1 group M subtypes and recombinants ranging from 1% of CRF01_AE sequences to 61% of subtype B. Including the proposed peripheral positions (339N, 386N, and 448N), the conservation of glycosylation of the 2G12 epitope drops to 1% for CRF01_AE up to 36% for subtype B. For subtype C and CRF01_AE, this low level of 2G12 conservation is not surprising as poor reactivity of 2G12 against them is well documented [27, 28, 60] due to the low levels of glycosylation at positions 295 and 332 in subtype C and CRF01_AE, respectively.

Binley and colleagues have clearly demonstrated the poor reactivity of 2G12 against both subtype C and CRF01_AE [27]. Examining the CRF01_AE nucleotide sequences from their study shows that two of the 2G12 core positions (295 and 392) are glycosylated in 100% of sequences (*n* = 9), and that the third core position (332) is not glycosylated in any of the sequences. However, 100% of the CRF01_AE sequences are glycosylated at 334, a position that has been suggested to compensate for loss of glycosylation at 332 in the 2G12 epitope [61]. Despite this, none of the CRF01_AE viruses were neutralized by 2G12 [27] suggesting that glycosylation at position 334 cannot replace 332 as part of the 2G12 epitope. Similarly, we have shown here that 96% of CRF01_AE sequences are glycosylated at position 334 instead of 332, and if 334 was truly compensating for 332 as part of the 2G12 epitope, one would expect CRF01_AE to be more sensitive to neutralisation than is observed [27, 28, 60].

The vast majority of subtype C viruses (84%, Figure 1) are not glycosylated at position 295, the position suggested to be responsible for the lack of subtype C neutralisation by 2G12 [28]. Gray and colleagues suggest that reintroduction of glycosylation at position 295 is not sufficient to restore 2G12 sensitivity, and that positions 442 and 448 are also responsible for conferring sensitivity to 2G12 [28]. This concurs with our examination of the sequence data from the study by Binley and colleagues where the only subtype C virus glycosylated at all positions of the 2G12 core epitope (295, 332, and 392) was not neutralized by 2G12 (most likely because it is not glycosylated at position 442). The remaining subtype C sequences (*n* = 11) show no glycosylation at position 295 with 10 of these being glycosylated at position 442. Consistent with previous work, none of these subtype C viruses were neutralized by 2G12 [27]. Only 4% of subtype C viruses show glycosylation at all of the core 2G12 epitope positions (295, 332, and 392) plus positions 442 and 448 suggesting that perhaps a very low number of wild-type subtype C viruses (containing the appropriate glycosylation pattern) may be neutralized by 2G12. While it has been previously suggested that 295 and 442 are mutually exclusive in subtype C [54], we see that 7% of subtype C sequences are glycosylated at both positions 295 and 442. The vast majority (77%) of subtype C sequences, however, show glycosylation at either position 295 or 442.

We observe that carbohydrates bound to positions 332 and 334 occupy overlapping three-dimensional space on the gp120 structure, as do positions 295 and 442. Therefore, it appears that while subtype C and CRF01_AE can evade neutralisation by 2G12 through the absence of glycosylation at essential positions, unique glycosylation at structurally proximal positions means that the protective qualities of the gp120 glycan shield are fully preserved.

Similarly, glycosylation at position 332 has been shown to significantly affect susceptibility to other recently described BCN antibodies [30–33] with Moore and colleagues observing that viruses that are glycosylated at position 334 are not susceptible to neutralisation by BCN antibodies described in their study [33]. Walker and colleagues suggest that positions 332 and/or 301 are important for neutralisation by at least six of the BCN antibodies described in their study [30] and this observation was supported by a structural study by Pejchal and colleagues [31]. While we observe that glycosylation at position 301 is relatively highly conserved across all of the subtypes and CRFs, the low level of glycosylation at position 332 in CRF01_AE means that it is rarely susceptible to neutralisation by any of the known BCN antibodies.

Thus, if fully broadly cross-clade neutralising synthetic anti-carbohydrate therapies are to be investigated, researchers should take into account the glycosylation anomalies observed in CRF01_AE and focus on amino acid positions where glycosylation is conserved across the entire spectrum of the HIV-1 group M subtypes and circulating recombinant forms. In order to identify such positions, we employed an approach to identify pairs and networks of glycosylated residues that show high levels of conservation across all of the HIV-1 group M subtypes and recombinant forms. Only five pairs of highly dependent glycosylated residues were identified as being conserved over all of the subtypes/CRFs (Figure 2). Of these five pairs only positions 88N and 241N exhibit similar levels of three-dimensional proximity (12.5 Å) to those of the 2G12 epitope (mean of 14.6 Å) when mapped onto the V3-containing gp120 core structure [57]. Similarly, positions 262N and 448N show high levels of glycosylation dependency in all but subtype C (Figure 2) and are proximal in the 3D structure. Whilst glycosylation at positions 262N and 448N is not identified as significant (shared in greater than 90% of sequences) for subtype C, the level of conservation (69%) is still greater than that of the 2G12 epitope in subtype B (61%). Similarly, networks of highly conserved glycosylated positions were identified for all subtypes/CRFs (Figure 2). While these highly conserved pairs/networks of glycosylated residues are evident, it is impossible to know whether these perceived dependencies are mutually dependent. Despite the extreme sequence diversity observed between HIV-1 group M subtypes/CRFs, glycosylation of these positions is highly conserved suggesting that there is a functional constraint on retaining glycosylation of these positions. These glycosylated positions form potential epitopes that are highly conserved across all HIV-1 group M subtypes and recombinants, and, as such, further investigation of potential epitopes for the development of BCN synthetic anti-carbohydrate therapeutics is warranted.

## 5. Conclusions

Here, we have shown that while N-linked glycosylation at many positions is highly conserved across the HIV-1 group M subtypes and CRFs, strong strain-specific glycosylation patterns exist and appear to affect the susceptibility broadly cross-neutralising antibodies. We suggest that alternate glycosylation patterns in some HIV strains allow evasion from neutralisation from BCN antibodies while still maintaining the overall protective qualities of the gp120 glycan shield. Further, we have identified highly conserved, structurally proximal, clusters of glycosylated positions that should be investigated further to explore their potential as epitopes against which BCN carbohydrate-binding therapies can be developed. 

## Supplementary Material

For each subtype/CRF, all available full length sequences were retrieved from the Los Alamos National Laboratory (LANL) HIV Sequence Database (http://www.hiv.lanl.gov/). Using the patient and isolate annotation, where available, multiple sequences representing single individuals were removed to ensure that the dataset representing each subtype/CRF was non-redundant. The fragment of the envelope gene that encodes for gp120 was excised from each full genome and separate datasets generated for each of the subtypes/CRFs studied. This supplementary table details all of the DNA sequences used in this study together with their designated subtype/CRF, genbank accession number, the country of origin and, where available, the year of sampling.

## Figures and Tables

**Table 1:** gp120 variable loop glycosylation. The average, median, range of lengths (number of amino acids), and number of predicted N-linked glycosylated positions for the gp120 variable loops of each of the HIV-1 group M subtypes and CRFs studied.

	A			B			C			D			AE			AG		
	AV	MED	RA	AV	MED	RA	AV	MED	RA	AV	MED	RA	AV	MED	RA	AV	MED	RA
	Length																	

V1/V2	68	68	57–92	68	68	46–85	68	67	51–89	65	67	57–82	69	69	55–85	66	65	53–89
V3	34	34	25–35	34	34	31–38	34	34	24–36	33	33	33–35	34	34	34–35	34	34	33–34
V4	31	30	22–40	31	31	15–41	27	27	17–39	30	30	21–37	27	27	18–39	31	31	24–38
V5	10	10	9–13	11	11	9–19	11	11	7–19	11	10.5	8–16	10	10	8–13	10	10	9–15

	Number of glycosylated positions																	

V1/V2	6	6	3–10	6	6	3–9	6	6	1–10	6	6	3–8	6	6	3–11	6	6	3–10
V3	1	1	NA	1	1	1-2	1	1	1-2	1	1	NA	1	1	1-2	1	1	1-2
V4	5	5	2–7	5	5	2–7	4	4	1–7	5	5	2–7	4	4	2–6	5	5	3–7
V5	2	2	1–3	1	1	1–3	1	1	1–4	2	2	1–3	2	2	1–3	2	2	1–3

AV: average, MED: median, RA: range.

**Table 2:** 2G12 conservation percentage of sequences representing each of the subtypes/CRFs exhibiting glycosylation at all positions in the conserved 2G12 core epitope (295N, 332N, and 392N) and the core epitope with peripheral glycans that have been suggested to be involved in 2G12 binding (339N, 386N, and 448N).

Subtype/CRF	Core epitope	Core + peripheral glycans
A	33%	27%
B	61%	36%
C	11%	5%
D	35%	23%
CRF01_AE	1%	1%
CRF02_AG	60%	35%
