# Supplementary material for: Conservation, Compensation, and Evolution of N-Linked Glycans in the HIV-1 Group M Subtypes and Circulating Recombinant Forms
Source: ISRN AIDS. 2012 Dec 19;2012:823605. doi: 10.5402/2012/823605 (PMC3765798; doi:10.5402/2012/823605)
Supplement: Supplementary file 1 — For each subtype/CRF, all available full length sequences were retrieved from the Los Alamos National Laboratory (LANL) HIV Sequence Database (http://www.hiv.lanl.gov/). Using the patient and isolate annotation, where available, multiple sequences representing single individuals were removed to ensure that the dataset representing each subtype/CRF was non-redundant. The fragment of the envelope gene that encodes for gp120 was excised from each full genome and separate datasets generated for each of the subtypes/CRFs studied. This supplementary table details all of the DNA sequences used in this study together with their designated subtype/CRF, genbank accession number, the country of origin and, where available, the year of sampling. [file 823605.f1.pdf]

Supplementary Table 1: All of the highly dependent glycosylated pairs identified for each of the subtypes/CRFs studied. Such pairs were identified as glycosylated together in greater than 90% of sequences representing that subtype/CRF. Percentages of sequences in which the pair was identified in are in brackets.

A	B	C	D	AE	AG
88 & 156 (94)	88 & 156 (93)	88 & 156 (94)	88 & 241 (91)	88 & 156 (95)	88 & 156 (96)
88 & 160 (94)	88 & 197 (94)	88 & 160 (92)	88 & 262 (95)	88 & 160 (91)	88 & 160 (98)
88 & 197 (95)	88 & 241 (94)	88 & 197 (96)	88 & 448 (95)	88 & 197 (95)	88 & 197 (98)
88 & 241 (93)	88 & 262 (98)	88 & 241 (95)	197 & 262 (93)	88 & 241 (93)	88 & 241 (100)
88 & 262 (97)	88 & 276 (95)	88 & 262 (97)	197 & 448 (93)	88 & 262 (97)	88 & 262 (100)
88 & 276 (95)	88 & 301 (90)	88 & 301 (95)	241 & 262 (95)	88 & 289 (94)	88 & 276 (93)
88 & 301 (91)	88 & 448 (93)	156 & 160 (90)	241 & 448 (95)	88 & 334 (93)	88 & 301 (90)
88 & 448 (95)	156 & 262 (93)	156 & 197 (94)	262 & 276 (91)	88 & 386 (96)	88 & 448 (95)
156 & 160 (93)	156 & 276 (90)	156 & 241 (93)	262 & 289 (91)	88 & 448 (96)	156 & 160 (95)
156 & 197 (94)	197 & 241 (90)	156 & 262 (94)	262 & 356 (91)	156 & 160 (91)	156 & 197 (95)
156 & 241 (90)	197 & 262 (94)	156 & 301 (92)	262 & 386 (93)	156 & 197 (95)	156 & 241 (96)
156 & 262 (94)	197 & 276 (92)	160 & 197 (92)	262 & 448 (100)	156 & 241 (94)	156 & 262 (96)
156 & 276 (93)	197 & 448 (90)	160 & 241 (91)	276 & 448 (91)	156 & 262 (97)	156 & 276 (90)
156 & 448 (93)	241 & 262 (95)	160 & 262 (93)	289 & 448 (91)	156 & 289 (93)	156 & 448 (92)
160 & 197 (94)	241 & 276 (91)	160 & 301 (91)	356 & 448 (91)	156 & 334 (93)	160 & 197 (96)
160 & 241 (90)	262 & 276 (95)	197 & 241 (96)	386 & 448 (93)	156 & 386 (95)	160 & 241 (98)
160 & 262 (94)	262 & 301 (90)	197 & 262 (97)		156 & 448 (96)	160 & 262 (98)
160 & 276 (93)	262 & 448 (93)	197 & 301 (95)		160 & 197 (91)	160 & 276 (92)
160 & 448 (93)	276 & 448 (90)	241 & 262 (96)		160 & 241 (91)	160 & 301 (90)
197 & 241 (91)		241 & 301 (94)		160 & 262 (93)	160 & 448 (93)
197 & 262 (95)		262 & 276 (90)		160 & 386 (91)	197 & 241 (98)
197 & 276 (94)		262 & 301 (96)		160 & 448 (92)	197 & 262 (98)
197 & 301 (90)				197 & 241 (94)	197 & 276 (92)
197 & 448 (94)				197 & 262 (97)	197 & 448 (93)
241 & 262 (93)				197 & 289 (93)	241 & 262 (100)
241 & 276 (91)				197 & 334 (93)	241 & 276 (93)
241 & 448 (91)				197 & 386 (95)	241 & 301 (90)
262 & 276 (95)				197 & 448 (96)	241 & 448 (95)
262 & 301 (91)				241 & 262 (95)	262 & 276 (93)
262 & 448 (95)				241 & 289 (91)	262 & 301 (90)
276 & 301 (90)				241 & 334 (91)	262 & 448 (95)
276 & 448 (94)				241 & 386 (93)	
301 & 448 (90)				241 & 448 (94)	
				262 & 276 (91)	
				262 & 289 (95)	
				262 & 334 (95)	
				262 & 386 (97)	
				262 & 448 (98)	
				289 & 334 (91)	
				289 & 386 (94)	
				289 & 448 (94)	
				334 & 386 (93)	
				334 & 448 (94)	
				386 & 448 (96)	
